# Safety and Efficacy of the Rechallenge of Immune Checkpoint Inhibitors After Immune-Related Adverse Events in Patients With Cancer: A Systemic Review and Meta-Analysis

**DOI:** 10.3389/fimmu.2021.730320

**Published:** 2021-09-27

**Authors:** Qing Zhao, Jianwei Zhang, Lingyi Xu, Huaxia Yang, Naixin Liang, Li Zhang, Fengchun Zhang, Xuan Zhang

**Affiliations:** ^1^ Department of Rheumatology and Clinical Immunology, Peking Union Medical College Hospital, Chinese Academy of Medical Sciences and Peking Union Medical College, Beijing, China; ^2^ The Ministry of Education Key Laboratory, National Clinical Research Center for Dermatologic and Immunologic Diseases, Beijing, China; ^3^ State Key Laboratory of Complex Severe and Rare Diseases, Peking Union Medical College Hospital, Chinese Academy of Medical Sciences and Peking Union Medical Collage, Beijing, China; ^4^ Key Laboratory of Carcinogenesis and Translational Research (Ministry of Education/Beijing), Department of Gastrointestinal Oncology, Peking University Cancer Hospital & Institute, Beijing, China; ^5^ Renal Division, Peking University First Hospital, Peking University Institute of Nephrology, Beijing, China; ^6^ Department of Thoracic Surgery, Peking Union Medical College Hospital, Chinese Academy of Medical Sciences and Peking Union Medical College, Beijing, China; ^7^ Department of Respiratory, Peking Union Medical College Hospital, Chinese Academy of Medical Sciences and Peking Union Medical College, Beijing, China; ^8^ Clinical Immunology Center, Medical Epigenetics Research Center, Chinese Academy of Medical Sciences and Peking Union Medical College, Beijing, China

**Keywords:** immune checkpoint inhibitors, immune-related adverse events, rechallenge, cancer, safety, efficacy

## Abstract

**Introduction:**

Little evidence exists on the safety and efficacy of the rechallenge of immune checkpoint inhibitors (ICIs) after immune-related adverse events (irAEs) in patients with cancer.

**Methods:**

We searched PubMed, Web of Science, Embase, and Cochrane for articles on ICI rechallenge after irAEs for systemic review and meta-analysis. The outcomes included the incidence and associated factors for safety and objective response rate (ORR) and disease control rate (DCR) for efficacy.

**Results:**

A total of 789 ICI rechallenge cases from 18 cohort studies, 5 case series studies, and 54 case reports were included. The pooled incidence of all-grade and high-grade irAEs after rechallenge in patients with cancer was 34.2% and 11.7%, respectively. Compared with initial ICI treatment, rechallenge showed a higher incidence for all-grade irAEs (OR, 3.81; 95% CI, 2.15–6.74; *p* < 0.0001), but similar incidence for high-grade irAEs (*p* > 0.05). Types of initial irAEs (pneumonitis and global irAEs) and cancer (non-small cell lung cancer and multiple cancer) recapitulated these findings. Gastrointestinal irAEs and time interval between initial irAEs and ICI rechallenge were associated with higher recurrence of high-grade irAEs (*p* < 0.05), whereas initial anti-PD-1/PD-L1 antibodies were associated with a lower recurrence (*p* < 0.05). Anti-PD-1/PD-L1 antibodies rechallenge was associated with a lower all-grade irAE recurrence (*p* < 0.05). The pooled ORR and DCR after rechallenge were 43.1% and 71.9%, respectively, showing no significant difference compared with initial ICI treatment (*p* > 0.05).

**Conclusions:**

ICI rechallenge after irAEs showed lower safety and similar efficacy outcomes compared with initial ICI treatment.

**Systematic Review Registration:**

PROSPERO, identifier CRD42020191405.

## Introduction

The development of immune checkpoint inhibitors (ICIs) targeting cytotoxic T-lymphocyte antigen-4 (CTLA-4), programmed cell death protein-1 (PD-1), or its ligand (PD-L1) is a milestone in cancer therapy. By interrupting the inhibitory signaling pathways of T-cell inhibition, ICIs can reinvigorate the T cells to recognize tumor antigens and recover the antitumor immune response (1). However, patients may experience immune-related adverse events (irAEs) because of the augmented immune response and unbalance of the immune system. As cancer patients exposed to ICIs increase in recent years, so does the number of irAEs. The incidence of grade 3 or 4 irAEs was approximately 14% after anti-PD-1 monotherapy (2), 23% after anti-CTLA-4 monotherapy (3), and 53% after combination therapy (4). IrAEs, especially grade 3 or 4 irAEs, needed timely identification and management. Most irAEs resolved after discontinuation of the ICIs and management with resuscitative efforts, systematic steroids, or other immunosuppressive agents (5, 6). However, whether patients should be rechallenged with ICIs after treatment of irAEs remains inconclusive.

Several recent studies demonstrated that ICI rechallenge is safe and reasonably efficacious by comparing the incidence of the initial and rechallenged irAEs and the objective response rate (ORR) of the initial and rechallenged ICIs (7, 8). Some studies concluded that ICI rechallenge might be an optional and promising treatment in select patients, and emphasized the importance of appropriate monitoring (9–13). Other studies, however, found no difference or even higher incidence of rechallenged irAEs than initial irAEs (14, 15). The latest guidelines suggest that partial grade 3 (including cardiovascular and neural events) and all grade 4 irAEs should discontinue ICI therapy (16). Nonetheless, the recommendations are mainly based on expert consensus and need to be backed up by more high-quality evidence. Besides, predisposing factors for the occurrence of rechallenged irAEs have recently been studied, but not decided yet (13). Therefore, a systematic review and meta-analysis of recent studies is required to evaluate the safety and efficacy of ICI rechallenge and reveal the related predisposing factors.

Herein, we conducted a systematic review and meta-analysis to explore the safety and efficacy of ICI rechallenge after initial irAEs in cancer patients. Furthermore, we investigated the association of the clinical factors of the patients with the safety and efficacy of ICI rechallenge.

## Methods

This study was conducted according to the Preferred Reporting Items for Systematic Reviews and Meta-Analyses (PRISMA) statement (see **Supplementary Material 1**) (17). We prospectively registered the protocol in PROSPERO International Register of Systematic Reviews (CRD42020191405).

### Literature Search Strategy

PubMed, Web of Science, Embase, and Cochrane databases were searched to identify relevant studies published from the database inception to June 9, 2020, with language confined to English. The key retrieval terms in the search strategy included cancer, tumor, neoplasm, immune checkpoint inhibitors (anti-PD-1, anti-PD-L1, anti-CTLA-4), specific ICI names (nivolumab, pembrolizumab, atezolizumab, durvalumab, avelumab, ipilimumab, cemiplimab), and some terms relevant to “rechallenge” (retreat, readministrate, restart, reinitiate, resume). The detailed search strategy is provided in **Supplementary Material 2: Table S1**. References of selected papers were also searched to identify additional studies.

### Inclusion and Exclusion Criteria

Inclusion and exclusion criteria of studies were established before the literature search. Studies have to fulfill the following criteria for eligibility: enrolled adult patients (aged over 18) and enrolled cancer patients who rechallenged ICIs after the initial irAEs. Studies not adhering to the inclusion criteria were excluded. Other exclusion criteria were as follows: patients concurrently treated with ICIs and other treatments (e.g., radical resection, radiation therapy, chemotherapy, or targeted therapy); no detailed information of irAEs or treatment outcomes of ICIs; non-clinical studies, review, systematic review, or conference abstract without exhaustive data; non-English articles; and no full-text original articles. Two researchers (QZ and LX) independently screened titles and abstracts of every search output to identify all studies that potentially met the inclusion criteria. Then, the full texts of all potentially eligible studies were read for further discrimination. The two researchers (QZ and LX) solved any discrepancies on study selection *via* discussion, and a third researcher (JZ) was consulted when necessary.

### Data Collection and Quality Assessment

All data were collected by two researchers (QZ and LX) independently in accordance with a predefined procedure. The following detailed characteristics of the study (cohort study, case series, and case report) were extracted: author, publication year, study design, cancer type, types of initial and rechallenge ICIs, rechallenge ratios, time interval between initial irAEs and ICI rechallenge, types and incidence of initial and rechallenged irAEs, ORR, and disease control rate (DCR) after rechallenge. Rechallenged irAEs included flared and novel irAEs after ICI rechallenge. The same two independent researchers (QZ and LX) assessed the methodological quality of all included studies using the Newcastle-Ottawa Scale (NOS) criteria (18), weighted as selection, comparability, and outcome. The scale ranges from 0 (poor methodological quality) to 9 (optimal methodological quality) points. Any discrepancies were solved *via* discussion or consultation with the third researcher (JZ).

### Outcomes

Safety assessment included incidence of all-grade rechallenged irAEs and incidence of high-grade rechallenged irAEs. The severity of irAEs was recorded as grade 1 to 5 based on version 5 of the Common Terminology Criteria for Adverse Events (CTCAE) of the National Cancer Institute (Bethesda, MD, USA). Grade ≥3 was considered as high-grade irAEs, while grade 1 or 2 was low-grade irAEs. Efficacy assessment included ORR and DCR after ICI rechallenge. ORR was defined as the rate of patients who had a complete response or partial response, while DCR was defined as the rate of patients who had a complete response, partial response, or stable disease.

### Data Analyses

We employed Review Manager 5.3 (Cochrane Community, London, UK) and SPSS 18.0 (SPSS Inc., Chicago, IL, USA) for statistical analyses and plotting. Synthesis of all-grade and high-grade rechallenged irAEs, ORR, and DCR was conducted *via* meta-analysis using pooled odds ratios (OR), with 95% confidence intervals (CIs) calculated *via* the Mantel–Haenszel model (19). The pooled incidence of all-grade and high-grade rechallenged irAEs and other available dividing factors was calculated *via* a meta-analysis of proportions. Since included studies in the meta-analysis were all retrospective studies, random-effects model with the Mantel–Haenszel model (19) was applied considering the significant heterogeneity, which was then proved by the *I*-squared (*I* ^2^) test. Heterogeneity was indicated as low (*I* ^2^ = 0% to 40%), moderate (*I* ^2^ = 40% to 70%), and substantial (*I* ^2^ = 70% to 100%). Predefined subgroup analysis was mainly conducted for accessible data including types of initial irAEs and cancer types. Moreover, we pooled individual-level cases for clinical factors of patients including age, gender, types and grade of initial irAEs, corticosteroid dosage, cancer type, types of initial and rechallenged ICIs, and time interval between initial irAEs and ICI rechallenge, additionally complementing analysis on the safety and efficacy of ICI rechallenge. Univariate and multivariate OR with 95% CIs were computed using a logistic regression model. Candidate factors with *p*-values <0.1 in the univariate analysis were included in the multivariate model. *p*-values were computed using an unpaired two-tailed Wald test. For sensitivity analysis, one study was sequentially omitted to judge the stability of the pooled results.

## Results

### Eligible Studies and Characteristics

**Figure 1:**
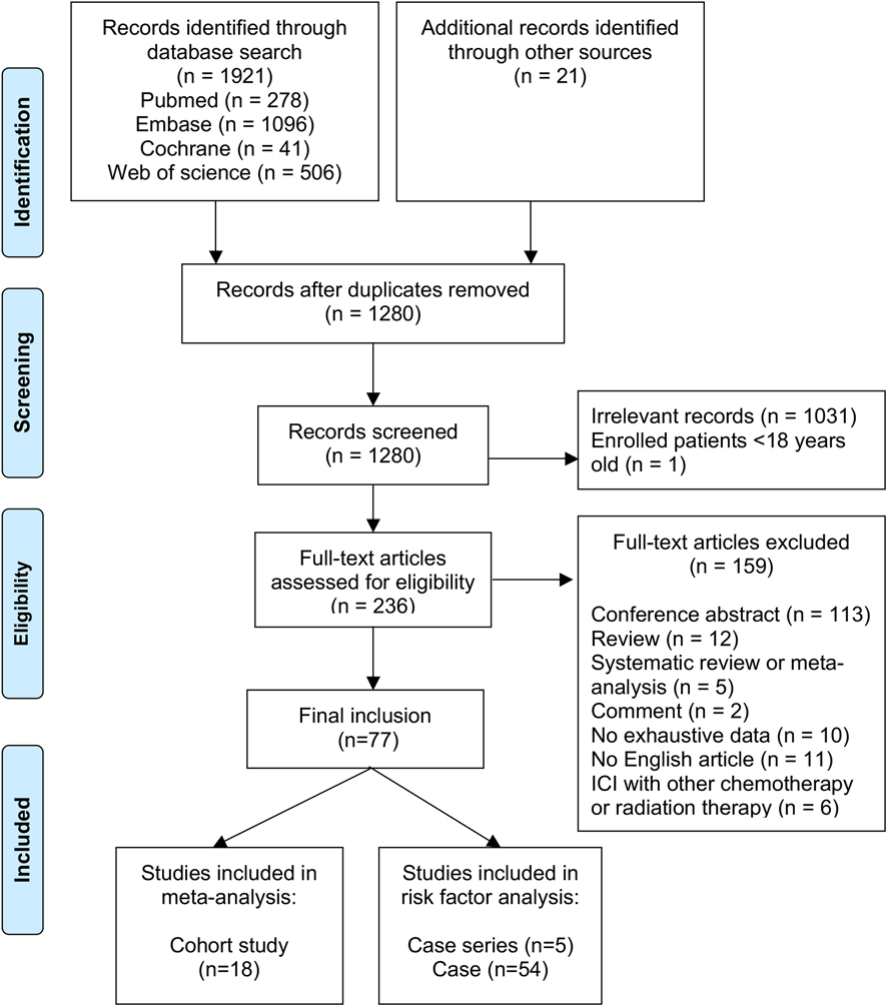
PRISMA 2009 flow diagram. “*n*” represents the number of studies.

Our literature search found 1,921 articles, and 21 additional articles were retrieved by searching the references of included studies. We ultimately reviewed the full texts of 236 articles after removing duplicates and screening titles and abstracts; of these, 77 studies comprising 788 individuals were enrolled for the present study (see **Figure 1**). The reasons for excluding the other 159 articles are listed in **Figure 1**.

Eighteen cohort studies comprising 691 patients were enrolled in the meta-analysis of safety and efficacy (see **Table 1** and **Supplementary Material 2: Table S2**). The median (range) number of patients enrolled was 31 (10–167) for safety and 19 (8–80) for efficacy. The included cancers were multiple cancer (seven studies, *n* = 372), melanoma (five studies, *n* = 189), non-small cell lung cancer (NSCLC) (five studies, *n* = 105), and colorectal cancer (one study, *n* = 25). Among the initial ICI treatment, anti-PD-1/PD-L1 monotherapy (191/416, 45.9%) was the most reported ICI therapy, followed by anti-CTLA-4 monotherapy (55/416, 13.2%) and combination therapy (170/416, 40.9%). Ten studies focused on global irAEs, and eight studies focused on specific irAEs, consisting of two for colitis/diarrhea, two for pneumonitis, and one each for neurological toxicity, pancreatic toxicity, hepatotoxicity, and acute kidney injury. Five hundred thirty-three patients reported initial irAE grades: low-grade (274/533, 51.4%) and high-grade (259/533, 48.6%). Anti-PD-1/PD-L1 monotherapy (449/691, 65.0%) was rechallenged more frequently than anti-CTLA-4 monotherapy (41/691, 5.9%) or combination therapy (27/691, 3.9%).

**Table 1:** Summary of study characteristics, safety, and efficacy of all included cohort studies.

Author	Study type	Cancer type	Initial ICIs type			Initial irAEs^a^				Rechallenge ratios	Type of rechallenged ICIs			Rechallenged irAEs				Disease response after rechallenge	
			CTLA-4	PD-1/PD-L1	Combinations	Type	Total irAEs	Low-grade irAEs	High-grade irAEs		CTLA-4	PD-1/PD-L1	Combination	Type	Total irAEs	Low-grade irAEs	High-grade irAEs	ORR	DCR
Abu-Sbeih (7)^b^	M	Multiple	47	79	41	Diarrhea and/or colitis	167	105	62	167/167	32/167	135/167	0/167	Diarrhea and/or colitis	57/167	51/167	6/167	NA	NA
Abu-Sbeih (20)	S	Multiple	627/2,279	1,434/2,279	218/2,279	Pancreatic injury	82/2,279	41/2,279	41/2,279	35/82	NA	NA	NA	Pancreatic injury	4/35	NA	NA	NA	NA
Amode (21)	S	Melanoma	82/82	0/82	0/82	NA	23/82	13/82	10/82	23/23	0/23	23/23	0/23	NA	14/23	10/23	4/23	5/23	8/23
Cortazar (22)^b,c^	M	Multiple	44	137	39	Acute kidney injury	138	NA	NA	31/138	NA	NA	NA	Acute kidney injury	7/31	NA	NA	NA	NA
Delyon (23)	S	Melanoma	NA	NA	NA	Diarrhea and/or colitis	25	NA	NA	11/25	2/11	8/11	1/11	Diarrhea and/or colitis	1/11	0/11	1/11	NA	NA
Dubey (24)	S	Multiple	186/1,834	1,215/1,834	433/1,834	Neurological	NA	NA	28/1,834	10/28	NA	NA	NA	Neurological	6/10	NA	NA	NA	NA
Fujita (15)	S	NSCLC	0/18	18/18	0/18	Global	NA	NA	NA	18/18	0/18	18/18	0/18	Global	NA	NA	NA	0/18	7/18
Fujita (9)	S	NSCLC	0/12	12/12	0/12	Global	NA	NA	NA	12/12	0/12	12/12	0/12	Global	NA	NA	NA	1/12	5/12
Koyauchi (25)	M	NSCLC	0/592	592/592	0/592	Pneumonitis	79/592	49/592	30/592	16/79	0/16	16/16	0/16	Pneumonitis	5/16	5/16	0/16	8/16	14/16
Menzies (26)^b^	M	Melanoma	NA	NA	NA	Global	67	9	58	67/67	0/67	67/67	0/67	NA	25/67	11/67	14/67	NA	NA
Miller (27)	S	Multiple	1,446/5,762	4,001/5,762	315/5,762	Hepatotoxicity	433/5,762	333/5,762	100/5,762	31/433	5/31	25/31	1/31	Hepatotoxicity	8/31	NA	NA	NA	NA
Morse (28)	M	Colorectal cancer	0/119	0/119	119/119	Global	67/119	38/119	29/119	25/67	0/25	0/25	25/25	Global	14/25	8/25	6/25	NA	NA
Mouri (29)	S	NSCLC	0/187	187/187	0/187	Global	49/187	34/187	15/187	21/49	0/21	21/21	0/21	Global	15/21	14/21	1/21	3/21	18/21
Naidoo (30)	M	Multiple	0/915	716/915	199/915	Pneumonitis	43/915	31/915	12/915	12/43	NA	NA	NA	Pneumonitis	3/12	3/12	0/12	NA	NA
Nomura (10)	S	Melanoma	0/8	8/8	0/8	Global	NA	NA	NA	8/8	2/8	6/8	0/8	Global	NA	NA	NA	2/8	5/8
Pollack (14)	M	Melanoma	0/80	0/80	80/80	Global	80/80	25/80	55/80	80/80	0/80	80/80	0/80	Global	40/80	26/80	14/80	56/80	71/80
Santini (11)	S	NSCLC	0/482	432/482	50/482	Global	68/482	35/482	33/482	38/68	0/38	38/38	0/38	NA	20/38	12/38	8/38	18/38	31/38
Williams (31)	S	Multiple	28/103	59/103	16/103	Global	103/103	79/103	24/103	86/103	NA	NA	NA	NA	4/86	NA	NA	NA	NA

CTLA-4, cytotoxic T-lymphocyte antigen-4; DCR, disease control rate; ICIs, immune checkpoint inhibitors; irAEs, immune-related adverse events; IS, immunosuppressant; M, multicenter retrospective study; NA, not applicable; NSCLC, non-small cell lung cancer; ORR, objective response rate; PD-1, programmed cell death protein-1; PD-L1, programmed cell death protein ligand-1; S, single-center retrospective study.

a Low-grade was considered as grades 1–2, and high-grade was considered as grade ≥3.

b No detailed information about all patients in the initial ICI treatment.

c Numbers in initial ICI type in this article denote all ICIs ever received.

Five case series and 54 case reports comprising 97 patients were enrolled for the analysis of factors associated with the safety and efficacy of ICI rechallenge (see **Supplementary Material 2: Table S3**). Melanoma, lung cancer, renal cancer, hematologic cancer, and other cancer types were reported in 41 (42.3%), 38 (39.2%), 2 (2.0%), 8 (8.2%), and 8 (8.2%) patients, respectively. Sixty-eight (70.1%) patients were initially treated with anti-PD-1/PD-L1 antibodies, 8 (8.2%) with anti-CTLA-4 antibodies, and 21 (21.6%) with combination. Anti-PD-1/PD-L1 monotherapy (83/97, 85.6%) was rechallenged more frequently than anti-CTLA-4 monotherapy (7/97, 7.2%) or combination therapy (7/97, 7.2%). Detailed demographic information was reported in 74 patients. In total, the median (range) age was 62 (30–87) years, and 47 (63.5%) were male. The initial irAEs were mainly respiratory [15 (20.3%)], gastrointestinal [12 (16.2%)], and hematologic [10 (13.5%)] irAEs. There were 27 (36.5%) low-grade, 36 (48.6%) high-grade, and 11 (14.9%) unknown grade irAEs. Nine (12.2%) patients were treated with low-/moderate-dose steroids, 49 (66.2%) with high-dose steroids, 7 (9.5%) with unknown-dose steroids, and 9 (12.2%) with unknown or no treatment.

### Safety

**Figure 2:**
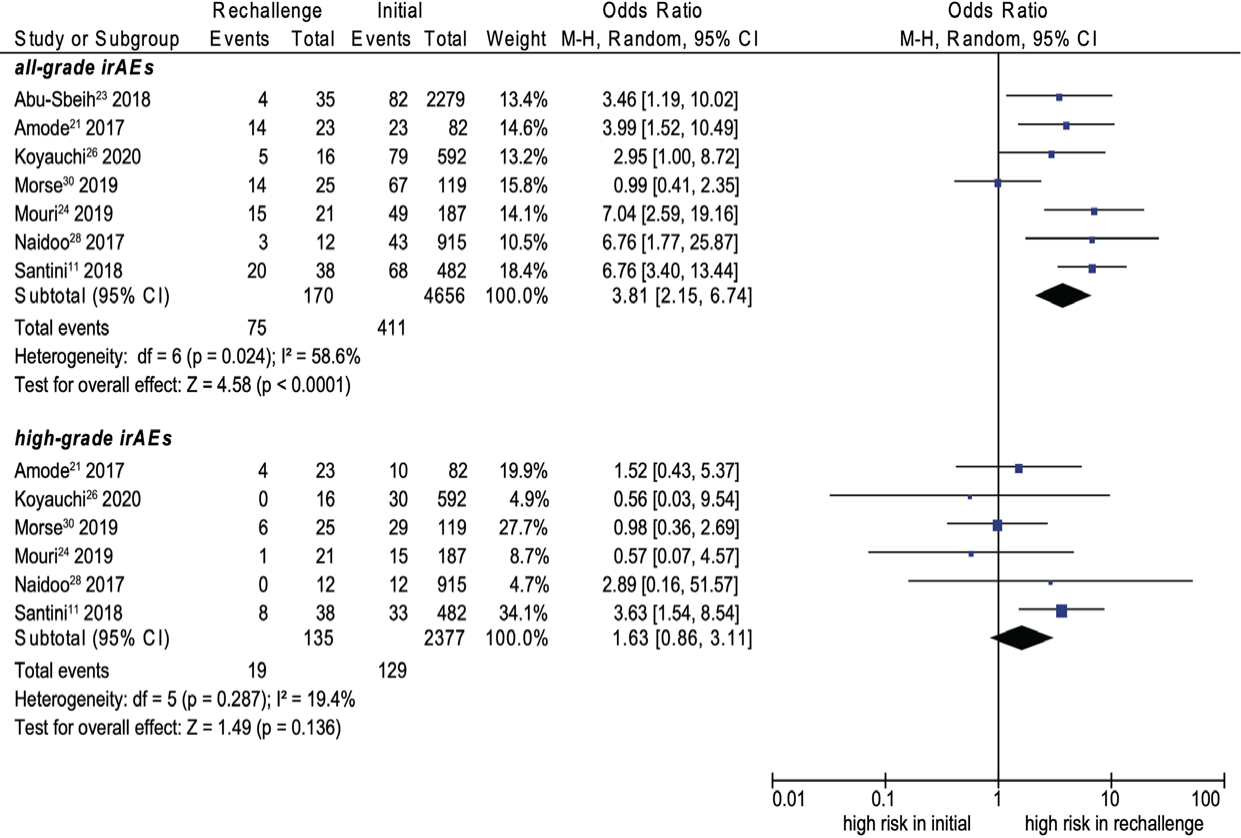
Forest plot (random-effects model) of the association between ICI rechallenge and all-grade or high-grade irAE occurrence after ICI rechallenge. CI, confidence interval; ICIs, immune checkpoint inhibitors; irAEs, immune-related adverse events; M-H, Mantel–Haenszel model. The sizes of the squares indicate the weight of the study. High-grade was considered as grade ≥3.

Fifteen cohort studies were included in the analysis of safety (7, 11, 14, 20–31). The recurrence rate of all-grade and high-grade irAEs were 34.2% and 11.7%, separately. ICI rechallenge was associated with a significantly higher incidence of all-grade irAEs than initial ICIs (OR, 3.81; 95% CI, 2.15–6.74; *p* < 0.0001; *I* ^2^ = 58.6%) (see **Figure 2**); however, no significant difference was noted for high-grade irAEs (OR, 1.63; 95% CI, 0.86–3.11; *p* = 0.136; *I* ^2^ = 19.4%) (see **Figure 2**).

**Figure 3:**
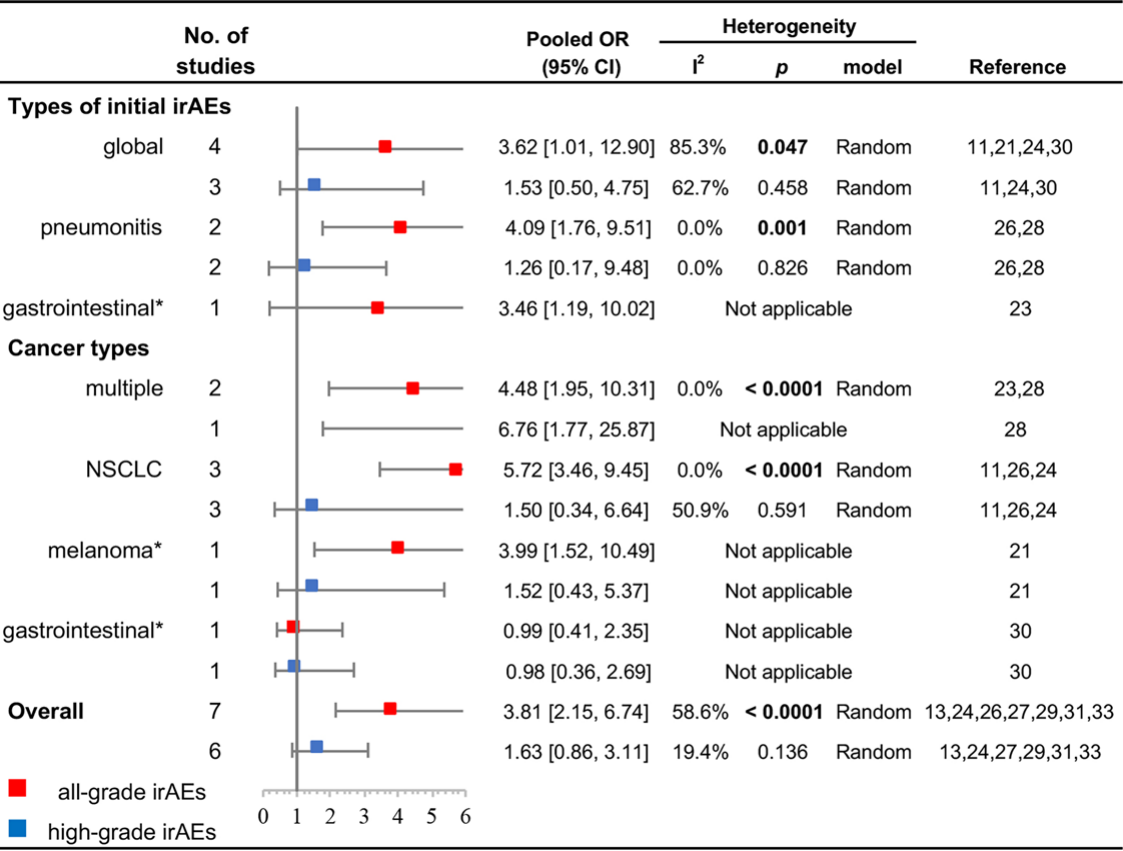
Subgroup analyses of the association between ICI rechallenge and all-grade or high-grade irAE occurrence after ICI rechallenge. CI, confidence interval; ICIs, immune checkpoint inhibitors; irAEs, immune-related adverse events; NSCLC, non-small cell lung cancer; OR, odds ratio. High-grade was considered as grade ≥3. “Global” indicates that the cohort included multiple irAEs. “Multiple” indicates that the cohort included patients with different cancer types. *The OR was directly presented without pooling because only one study was available.

The results of subgroup analyses for types of initial irAEs and cancer types are displayed in **Figure 3**. Initial pneumonitis was associated with a higher all-grade recurrence (OR, 4.09; 95% CI, 1.76–9.51; *p* = 0.001; *I* ^2^ = 0.0%), but not with a higher high-grade recurrence (OR, 1.26; 95% CI, 0.17–9.48; *p* = 0.826; *I* ^2^ = 0.0%). Initial global irAEs were also associated with a higher all-grade recurrence (OR, 3.62; 95% CI, 1.01–12.90; *p* = 0.047; *I* ^2^ = 85.3%); however, no significant difference was noted for high-grade recurrence (OR, 1.53; 95% CI, 0.50–4.75; *p* = 0.458; *I* ^2^ = 62.7%). Patients with NSCLC had a higher incidence of all-grade rechallenged irAEs (OR, 5.72; 95% CI, 3.46–9.45; *p* < 0.0001; *I* ^2^ = 0.0%), but no significant difference existed in high-grade irAEs (OR, 1.50; 95% CI, 0.34–6.64; *p* = 0.591; *I* ^2^ = 50.9%). Cohorts enrolled with multiple cancers also showed a significantly higher incidence of all-grade irAEs in ICI rechallenge (OR, 4.48; 95% CI, 1.95–10.31; *p* < 0.0001; *I* ^2^ = 0.0%).

Further analysis revealed that the recurrence rate of all-grade irAEs was not significantly different in different rechallenged ICIs (*χ* ^2^ = 0.800, *p* = 0.670, *df* = 2). Specifically, for patients initially treated with anti-PD-1/PD-L1 antibodies, anti-CTLA-4 antibodies rechallenge had a significantly higher incidence of all-grade irAEs than anti-PD-1/PD-L1 antibodies rechallenge (*p* = 0.040). However, for patients initially treated with anti-CTLA-4 antibodies or combination, no significant difference existed in the incidence of all-grade irAEs in different rechallenged ICIs (initial anti-CTLA-4 antibodies: *χ* ^2^ = 0.248, *p* = 0.618, *df* = 1; initial combination: *χ* ^2^ = 0.391, *p* = 0.532, *df* = 1) (see **Supplementary Material 2: Tables S4, S5**).

### Efficacy

**Figure 4:**
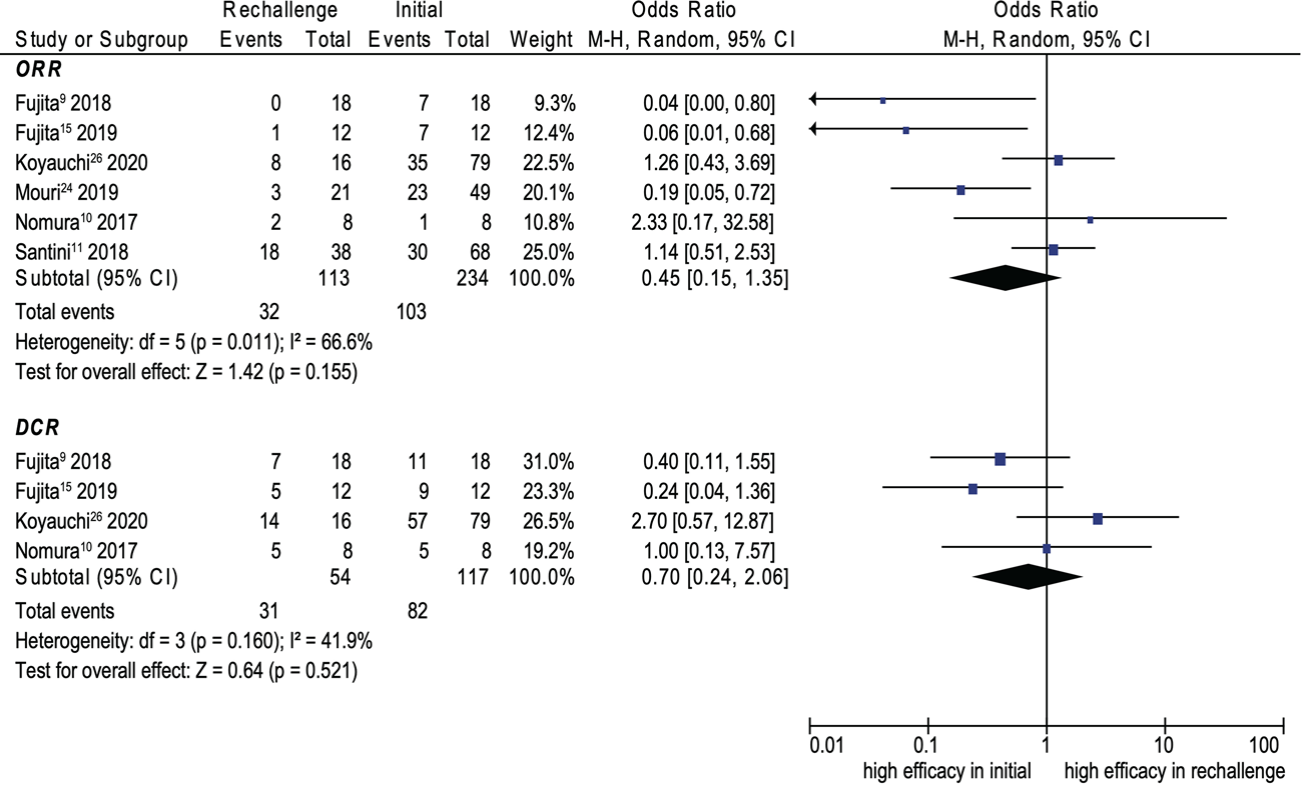
Forest plot (random-effects model) of the association between ICI rechallenge and ORR or DCR after ICI rechallenge. CI, confidence interval; DCR, disease control rate; ICIs, immune checkpoint inhibitors; irAEs, immune-related adverse events; M-H, Mantel–Haenszel model; ORR, objective response rate. The sizes of the squares indicate the weight of the study.

**Figure 5:**
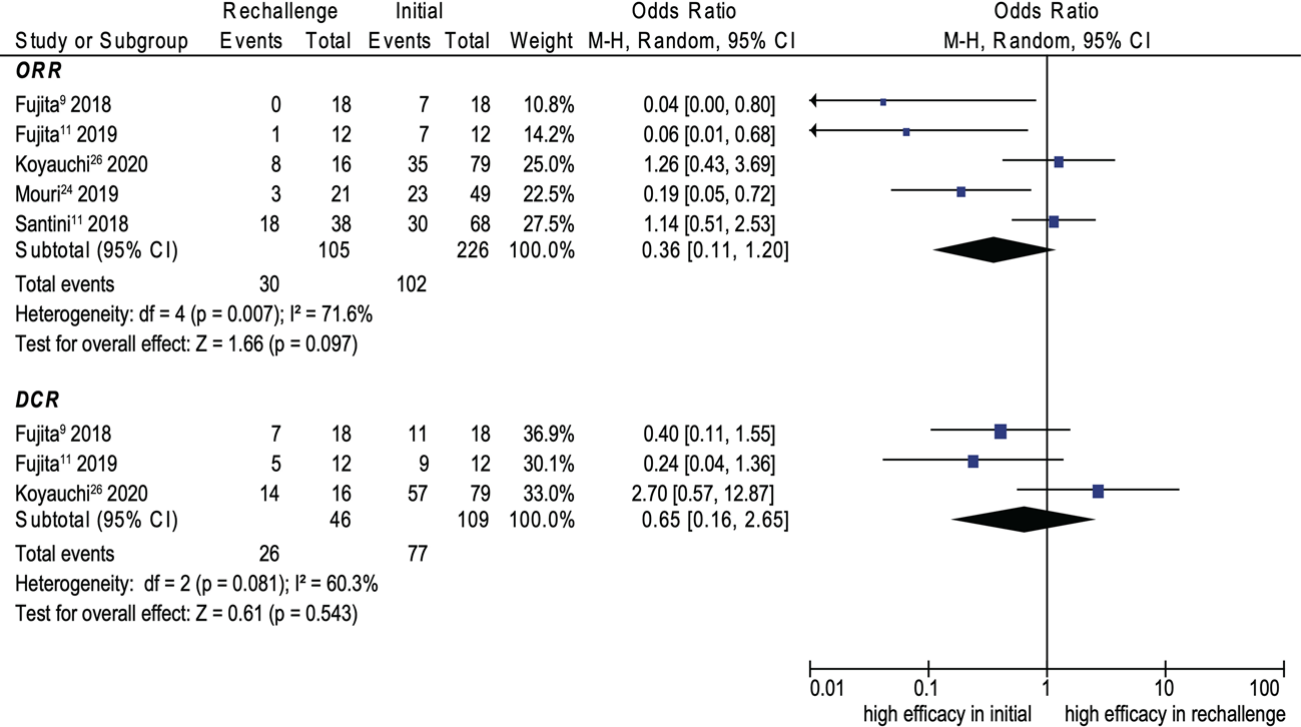
Forest plot (random-effects model) of the association between ICI rechallenge and ORR or DCR after ICI rechallenge in patients with NSCLC. CI, confidence interval; DCR, disease control rate; ICIs, immune checkpoint inhibitors; irAEs, immune-related adverse events; M-H, Mantel–Haenszel model; NSCLC, non-small cell lung cancer; ORR, objective response rate. The sizes of the squares indicate the weight of the study.

Eight cohort studies were included in the analysis of efficacy (9–11, 14, 15, 21, 25, 29). The pooled ORR and DCR of ICI rechallenge were 43.1% and 73.6%, respectively. No significant difference was noted between initial ICI treatment and ICI rechallenge for ORR (OR, 0.45; 95% CI, 0.15–1.35; *p* = 0.155; *I* ^2^ = 66.6%) (see **Figure 4**). Similarly, no significant difference was noted between initial ICI treatment and ICI rechallenge for DCR (OR, 0.70; 95% CI, 0.24–2.06; *p* = 0.521; *I* ^2^ = 41.9%) (see **Figure 4**). Further pooled analysis revealed that compared with initial ICI treatment, ICI rechallenge in patients with NSCLC has no significant difference for ORR (OR 0.36; 95% CI, 0.11–1.20; *p* = 0.097; *I* ^2^ = 71.6%) and DCR (OR, 0.65; 95% CI, 0.16–2.65, *p* = 0.543; *I* ^2^ = 60.3%) (see **Figure 5**).

### Factors Associated With the Safety and Efficacy of ICI Rechallenge

**Table 2** shows the factors associated with the occurrence of all-grade and high-grade rechallenged irAEs. For high-grade rechallenged irAEs, univariate analysis showed that gastrointestinal irAEs (OR, 6.81; 95% CI, 1.58–29.26; *p* = 0.010) and time interval between initial irAEs and ICI rechallenge (OR, 1.02; 95% CI, 1.00–1.05; *p* = 0.031) were associated with a higher recurrence, whereas initial anti-PD-1/PD-L1 antibodies were associated with a lower recurrence (OR, 0.26; 95% CI, 0.07–0.99; *p* = 0.049). For all-grade rechallenged irAEs, anti-PD-1/PD-L1 antibodies rechallenge was associated with a lower recurrence (OR, 0.28; 95% CI, 0.08–0.94; *p* = 0.039). Factors selected from the univariate analysis (*p* < 0.1) for multivariate analysis showed no significance for both high-grade and all-grade rechallenged irAEs (*p* > 0.05).

**Table 2:** Factors associated with occurrence of all-grade and high-grade rechallenged irAEs.

	High-grade irAEs						All-grade irAEs					
	*n* (total numbers, %)^a^		Univariate analysis		Multivariate analysis		*n* (total numbers, %)^a^		Univariate analysis		Multivariate analysis	
	Recurrence	Non-recurrence	OR (95% CI)	*p*	OR (95% CI)	*p*	Recurrence	Non-recurrence	OR (95% CI)	*p*	OR (95% CI)	*p*
Age	56.91 ± 10.13	63.11 ± 12.26	0.96 (0.91, 1.01)	0.126	/	/	59.86 ± 12.47	62.69 ± 12.16	0.98 (0.94, 1.02)	0.333	/	/
Gender (male)	8 (11, 72.7%)	35 (55, 63.6%)	0.66 (0.16, 2.76)	0.565	/	/	19 (29, 65.5%)	28 (45, 62.2%)	0.87 (0.33, 2.30)	0.774	/	/
Types of initial irAEs												
Gastrointestinal^b^	5 (11, 45.5%)	6 (55, 10.9%)	6.81 (1.58, 29.26)	**0.010**	1.25 (0.16, 9.75)	0.829	6 (29, 20.7%)	6 (45, 13.3%)	1.70 (0.49, 5.88)	0.405	/	/
Nephritic	1 (11, 9.1%)	2 (55, 3.6%)	2.65 (0.22, 32.08)	0.444	/	/	1 (29, 3.4%)	2 (45, 4.4%)	0.77 (0.07, 8.87)	0.832	/	/
Hematologic	2 (11, 18.2%)	7 (55, 12.7%)	1.52 (0.27, 8.55)	0.632	/	/	3 (29, 10.3%)	7 (45, 15.6%)	0.63 (0.15, 2.65)	0.525	/	/
Neurologic	1 (11, 9.1%)	8 (55, 14.5%)	0.59 (0.07, 5.24)	0.634	/	/	6 (29, 20.7%)	4 (45, 8.9%)	2.67 (0.68, 10.46)	0.158	/	/
Endocrine	0 (11, 0.0%)	7 (55, 12.7%)	NA	0.999	/	/	1 (29, 3.4%)	7 (45, 15.6%)	0.19 (0.02, 1.67)	0.135	/	/
Respiratory	2 (11, 18.2%)	10 (55, 18.2%)	1.00 (0.19, 5.36)	1.000	/	/	7 (29, 24.1%)	8 (45, 17.8%)	1.47 (0.47, 4.62)	0.508	/	/
Ocular	0 (11, 0.0%)	2 (55, 3.6%)	NA	0.999	/	/	2 (29, 6.9%)	1 (45, 2.2%)	3.26 (0.28, 37.69)	0.344	/	/
Rheumatologic	0 (11, 0.0%)	8 (55, 14.5%)	NA	0.999	/	/	3 (29, 10.3%)	5 (45, 11.1%)	0.92 (0.20, 4.20)	0.917	/	/
Dermatologic	0 (11, 0.0%)	5 (55, 9.1%)	NA	0.999	/	/	0 (29, 0.0%)	5 (45, 11.1%)	NA	0.999	/	/
Initial irAE grade^c^			2.96 (0.57, 15.49)	0.198	/	/			0.71 (0.26, 1.96)	0.504	/	/
Low-grade	2 (10, 20.0%)	20 (47, 42.6%)					12 (25, 48.0%)	15 (38, 39.5%)				
High-grade	8 (10, 80.0%)	27 (47, 57.4%)					13 (25, 52.0%)	23 (38, 60.5%)				
Initial corticosteroid dosage^d^			NA	0.999	/	/			1.27 (0.28, 5.68)	0.757	/	/
Low/moderate dose	0 (8, 0.0%)	9 (44, 20.5%)					3 (22, 13.6%)	6 (36, 16.7%)				
High dose	8 (8, 100.0%)	35 (44, 79.5%)					19 (22, 86.4%)	30 (36, 83.3%)				
Cancer type												
Melanoma	6 (11, 54.5%)	29 (55, 52.7%)	1.08 (0.29, 3.95)	0.912	/	/	18 (29, 62.1%)	23 (45, 51.1%)	1.57 (0.61, 4.05)	0.356	/	/
Lung	3 (11, 27.3%)	12 (55, 21.8%)	1.34 (0.31, 5.86)	0.694	/	/	5 (29, 17.2%)	10 (45, 22.2%)	0.73 (0.22, 2.40)	0.604	/	/
Renal	1 (11, 9.1%)	1 (55, 1.8%)	5.40 (0.31, 93.61)	0.247	/	/	2 (29, 6.9%)	0 (45, 0.0%)	NA	0.999	/	/
Hematologic	0 (11, 0.0%)	6 (55, 10.9%)	NA	0.999	/	/	2 (29, 6.9%)	6 (45, 13.3%)	0.48 (0.09, 2.57)	0.392	/	/
Initial ICI types												
PD-1/PD-L1^b^	4 (11, 36.4%)	38 (55, 69.1%)	0.26 (0.07, 0.99)	**0.049**	0.46 (0.06, 3.37)	0.448	14 (29, 48.3%)	31 (45, 68.9%)	0.42 (0.16, 1.11)	0.079	0.57 (0.19, 1.71)	0.312
CTLA-4	3 (11, 27.3%)	4 (55, 7.3%)	4.78 (0.90, 25.46)	0.067	1.16 (0.11, 12.40)	0.904	5 (29, 17.2%)	3 (45, 6.7%)	2.92 (0.64, 13.29)	0.167	/	/
Combination	4 (11, 36.4%)	13 (55, 23.6%)	1.85 (0.47, 7.32)	0.383	/	/	10 (29, 34.5%)	11 (45, 24.4%)	1.63 (0.58, 4.53)	0.352	/	/
Time interval between initial irAEs and ICI rechallenge (weeks)^b^	24.0 (11.5, 100.0)	10.0 (4.0, 25.0)	1.02 (1.00, 1.05)	**0.031**	1.02 (1.00, 1.04)	0.089	13.0 (5.0, 43.0)	14.0 (5.0, 29.0)	1.01 (0.99, 1.03)	0.295	/	/
Rechallenged ICI types												
PD-1/PD-L1^b^	7 (11, 63.6%)	47 (55, 85.5%)	0.30 (0.07, 1.26)	0.099	0.43 (0.06, 2.91)	0.387	20 (29, 69.0%)	40 (45, 88.9%)	0.28 (0.08, 0.94)	**0.039**	0.56 (0.10, 3.25)	0.517
CTLA-4	2 (11, 18.2%)	4 (55, 7.3%)	2.83 (0.45, 17.83)	0.267	/	/	5 (29, 17.2%)	2 (45, 4.4%)	4.48 (0.81, 24.87)	0.086	2.24 (0.23, 21.42)	0.485
Combination	2 (11, 18.2%)	4 (55, 7.3%)	2.83 (0.45, 17.83)	0.267			4 (29, 13.8%)	3 (45, 6.7%)	2.24 (0.46, 10.84)	0.316		

CI, confidence interval; CTLA-4, cytotoxic T-lymphocyte antigen-4; ICIs, immune checkpoint inhibitors; irAEs, immune-related adverse events; NA, not applicable; OR, odds ratio; PD-1, programmed cell death protein-1; PD-L1, programmed cell death protein ligand-1.

a Qualitative variables were reported as n (total numbers, %), and quantitative variables were reported as mean ± standard deviation (SD) or a median with interquartile range (IQR).

b Clinical factors of patients with p-values <0.05.

c Initial irAE grade: low-grade was considered as grades 1–2, and high-grade was considered as grade ≥3.

d Low-dose was considered as “prednisone ≤ 7.5 mg/day” or “methylprednisolone ≤ 6 mg/day”; moderate-dose was considered as “7.5 mg/day < prednisone ≤ 30 mg/day” or “6 mg/day < methylprednisolone ≤ 24 mg/day”; high-dose was considered as “prednisone > 30 mg/day” or “methylprednisolone > 24 mg/day.” All significant p values are emphasized in bold.

Univariate analysis for ORR and DCR after ICI rechallenge showed that no clinical factors of patients were found significant (*p* < 0.05) and multivariate analysis was not applicable (see **Supplementary Material 2: Table S6**).

### Sensitivity Analysis

In the sensitivity analysis, the pooled results for all-grade irAEs, high-grade irAEs, ORR, and DCR remained stable, regardless of which study was deleted, which indicates the robust association (see **Supplementary Material 2: Figure S1**).

## Discussion

To our knowledge, this study represents the largest and most comprehensive analysis of the safety and efficacy of ICI rechallenge. The main conclusions drawn based on our results are as follows:

-ICI rechallenge was associated with a higher incidence of all-grade irAEs than initial ICIs; however, the incidence for high-grade irAEs was not significantly different.
-No significant difference in efficacy existed after ICI rechallenge compared with initial ICIs.
-Gastrointestinal irAEs and the time interval between initial irAEs and ICI rechallenge were factors associated with a higher recurrence rate of high-grade irAEs, whereas initial anti-PD-1/PD-L1 antibodies were associated with a lower recurrence rate.
-Anti-PD-1/PD-L1 antibodies rechallenge was a factor associated with a lower recurrence rate of all-grade irAEs.

The incidence of all-grade irAEs after ICI rechallenge in our study was 34.2%, reproduced by other studies showing all-grade rechallenged irAEs of 27.5%–55% (8, 32). We found a higher incidence of all-grade irAEs in the rechallenged group compared with the initial group. Abou Alaiwi et al. conducted a multicenter retrospective study involving 499 patients with metastatic renal cell carcinoma and found that irAEs occurred in 50% of the patients rechallenged with ICIs, higher than 16% in initial ICIs (33). No significant differences were noted between ICI rechallenge and the initial ICIs for high-grade irAEs. The possible reasons for the similar occurrence of high-grade irAEs were as follows. First, ICI discontinuation was recommended for most high-grade irAEs since they have already constituted the contraindication of rechallenge (16). Second, closer monitoring and earlier management of irAEs after ICI rechallenge were performed. ICI rechallenge needs appropriate monitoring and standard treatment algorithms to identify and treat toxic effects. Besides, more research is warranted to further explore the safety profile of ICI rechallenge.

Our data showed that for patients initially treated with anti-PD-1/PD-L1 antibodies, anti-CTLA-4 antibodies rechallenge had a significantly higher incidence of all-grade irAEs than anti-PD-1/PD-L1 antibodies rechallenge. Dolladille et al. found that initial anti-CTLA-4 monotherapy was associated with a higher incidence of the same irAEs in ICI rechallenge (13). Studies have shown that anti-CTLA-4 antibodies suppress the initial priming events in T-cell activation, while anti-PD-1 antibodies inhibit the effector phase of T cells in the periphery (34). Anti-CTLA-4 antibodies reactivate immune function at an earlier stage of T-cell activation compared with anti-PD-1 antibodies, which might directly disrupt the central tolerance and explain the higher recurrence rate of irAEs. Besides, a pharmacodynamics study has indicated more than 70% of PD-1 molecules on peripheral blood T cells were occupied for more than 2 months after being treated with anti-PD-1 antibodies (35). Therefore, switching from anti-PD-1 antibodies to anti-CTLA-4 antibodies may be equivalent to giving these antibodies combined, leading to a possibly higher irAE recurrence.

The ORR and DCR of ICI rechallenge were 43.1% and 71.9%, respectively. Our data were consistent with studies showing an ORR of 23%–37.5% and a DCR of 48.4%–75.0% by evaluating patients with melanoma after rechallenge (36, 37) and an ORR of 23%–44% and a DCR of 64% by evaluating patients with renal cancer after rechallenge (8, 33). We found no significant difference for ORR and DCR between ICI rechallenge and initial ICIs. For the included studies, only two and one studies show similar ORR and DCR (10, 11, 25), respectively. More studies are needed to solve the discrepancy of efficacy after ICI rechallenge. Since cancer types influenced the efficacy of ICI rechallenge inherently, we further pooled ORR and DCR in patients with NSCLC. No significant differences for ORR and DCR between ICI rechallenge and initial ICIs were noted, which implied similar efficacy. However, considering the limited sample size of the pooled analysis, large-scale prospective studies are needed to confirm the limited effect of ICI rechallenge in various primary cancer types.

Initial gastrointestinal irAEs, including colitis, diarrhea, and hepatitis, were associated with a higher incidence of high-grade rechallenged irAEs. Dolladille et al. also reported that colitis and hepatitis were associated with a higher irAE recurrence after ICI rechallenge (13). Besides, gastrointestinal irAEs are the most common adverse events in initial ICI treatment (38). However, the underlying pathophysiology is still unknown. A possible explanation is the central role of regulatory cells and receptors, which are the target of ICIs, in maintaining the gastrointestinal barrier. Another explanation might be the intestinal microbiota. Microbial epitopes important for host protection to GI infection may overlap with tumor neoantigens (39). Our study demonstrated that the grade of initial irAEs did not predict rechallenged irAEs. Several prior studies also showed no association between the severity of initial irAEs and the recurrence rate of irAEs (13, 14). However, Kartolo et al. found that initial grade 3 irAEs were a risk factor for rechallenged irAEs (40). Limited studies focused on the association between timing of ICI rechallenge and the outcomes of ICI rechallenge. Our study observed that a longer time interval between initial irAEs and ICI rechallenge was associated with a higher recurrence rate of high-grade irAEs, indicating that the clinicians should be aware of their timing of ICI rechallenge. The types of ICIs were also questions that clinicians should consider. Dolladille et al. found that initial anti-CTLA-4 antibodies were associated with a higher irAE recurrence rate (13). Our study found that the initial and rechallenged anti-PD-1/PD-L1 antibodies showed a lower recurrence rate of high-grade and all-grade irAEs, respectively.

However, several limitations of our study should be noted. First, although we have tried to include the best evidence to date, no publications included in our study were prospective studies, raising concerns for the quality of evidence. Second, we used ORR and DCR as values for efficacy outcomes, while meta-analysis for OS and PFS was not performed since these data were not systematically reported in the recruited studies. Finally, the association between initial irAE grades, or initial corticosteroid dosage and outcomes of ICI rechallenge, in which clinical practice is more interested, could not be evaluated using data from cohort studies, but data from case series and case reports. Thus, these results should be interpreted with caution. More well-designed studies are warranted to evaluate the safety and efficacy of ICI rechallenge and reveal the predictive factors.

## Conclusions

Our study found that ICI rechallenge after irAEs was associated with lower safety and similar efficacy outcomes compared with initial ICI treatment in cancer patients. Further large-scale prospective studies are warranted to confirm our discoveries.

## Data Availability Statement

The original contributions presented in the study are included in the article/**Supplementary Material**. Further inquiries can be directed to the corresponding authors.

## Author Contributions

HY and NL designed the study protocol. QZ, JZ, and LX retrieved and selected the articles, analyzed and interpreted the data, and wrote the manuscript. HY and NL solved all disagreements and revised the manuscript. LZ, XZ, and FZ supervised the study. All authors contributed to the article and approved the submitted version.

## Funding

This work was supported by the CAMS Innovation Fund for Medical Sciences (CIFMS) (grant numbers 2020-I2M-C&T-B-011, 2020-I2M-C&T-A-003), National Natural Science Fund (grant number 81801633), CSCO Pilot Oncology Research Fund (grant number Y-2019AZMS-0452), CSCO-MSD fund (grant number Y-MSD2020-0270), and Wu Jieping Medical Foundation Precision Treatment for Thoracic and Abdominal Cancer Fund (grant number 320.6750.19092-43).

## Conflict of Interest

The authors declare that the research was conducted in the absence of any commercial or financial relationships that could be construed as a potential conflict of interest.

## Publisher’s Note

All claims expressed in this article are solely those of the authors and do not necessarily represent those of their affiliated organizations, or those of the publisher, the editors and the reviewers. Any product that may be evaluated in this article, or claim that may be made by its manufacturer, is not guaranteed or endorsed by the publisher.
